# SUPPORTIVE HOME FEATURES AS PROACTIVE/REACTIVE ASSOCIATES OF DAILY FUNCTIONING IN OLDER ADULTS

**DOI:** 10.1093/geroni/igae098.0008

**Published:** 2024-12-31

**Authors:** Daniel Barbakoff, Kristen Glennie, Bernard A Steinman

**Affiliations:** Weill Cornell Medicine, New York, New York, United States; Colorado State University, Fort Collins, Colorado, United States; University of Wyoming, Laramie, Wyoming, United States

## Abstract

Supportive home features (SHF) include attributes of living spaces meant to help people overcome physical limitations that may be barriers to living independently. Evidence suggests that SHFs, including home modifications (HM), may help older adults to age in place at home. However, real-life implementation of HMs is not fully understood. The purpose of this study was to clarify associations between functioning in daily-living activities and the presence of SHFs. We analyzed two waves (2011 and 2015) of the National Health and Aging Trends Study (NHATS) to examine the directionality of this relationship. SHFs were operationalized using indicators of home accessibility (e.g., presence of a bedroom, kitchen, and full bathroom with a shower or tub on the same floor). HMs were operationalized based on whether bathroom fixtures were modified to accommodate disability with features such as grab bars. Daily activities were assessed using measures of activities of daily living (ADL; e.g., dressing) and instrumental ADLs (IADL; e.g., preparing a meal). Changes in ADL/IADL functioning and SHF/HM variables were assessed over the 5-year period. Regression models also included covariates for sociodemographics, chronic conditions, mobility functioning, and participation. Results show that, whereas ADL limitations statistically predict SHF/HM outcomes, the implementation of SHF/HMs does not predict any ADL/IADL outcomes. These findings suggest that in real life, older adults implement SHF/HMs reactively in response to functional needs, as opposed to planning proactively to meet needs in the future.

